# *MgpB* Types among *Mycoplasma genitalium* Strains from Men Who Have Sex with Men in Berlin, Germany, 2016–2018

**DOI:** 10.3390/pathogens9010012

**Published:** 2019-12-20

**Authors:** Roger Dumke, Marcos Rust, Tobias Glaunsinger

**Affiliations:** 1 TU Dresden, Institut für Medizinische Mikrobiologie und Hygiene, Fetscherstrasse 74, 01307 Dresden, Germany; 2 Infektiologie Ärztezentrum Seestrasse, Seestrasse 64, 13347 Berlin, Germany; marcos.rust@infektiologie-seestrasse.de; 3 Praxis Prenzlauer Berg, Danziger Strasse 78b, 10405 Berlin, Germany; glaunsinger@praxis-prenzlauer-berg.de

**Keywords:** sexually transmitted infection, *Mycoplasma genitalium*, genotyping, MgPa, antimicrobial resistance

## Abstract

*Mycoplasma genitalium* is a cell wall-less bacterium causing urethritis and other sexually transmitted diseases. Despite a strongly conserved genome, strains in clinical samples can be typed by different methods. To obtain data from the risk population of men having sex with men, we analyzed the typing region in the gene coding for the MgpB adhesin of *M. genitalium* first in 163 and 45 follow-up samples among patients of two specialized practices in Berlin, Germany. Strains belong to 43 different *mgpB* types emphasizing the diversity of the genome region. With respect to 133 types previously described, 27 new types were found. However, the majority of strains (64.4%) were assigned to types 4, 6, 113, and 108, respectively. A correlation between *mgpB* type and the occurrence of mutations associated with macrolide and quinolone resistance was not demonstrated. Investigation of follow-up samples from 35 patients confirmed the same *mgpB* and, additionally, *MG_309* types in 25 cases. In 10 cases, differences between types in subsequent samples indicated an infection with a genetically different strain in the period between samplings. *MgpB*/*MG_309* typing is a useful method to compare *M. genitalium* strains in samples of individual patients as well as those circulating in different populations

## 1. Introduction

Members of the Mollicutes class are bacteria that lack the classical cell wall. Among them, some species are important for human health because they colonize mucosal surfaces and cause long-lasting, common but mostly self-limiting infections. The extremely slow-growing species *Mycoplasma genitalium* is a sexually transmitted pathogen that causes urethritis in men and is associated with cervicitis and pelvic inflammatory disease in women [1,2]. The prevalence of *M. genitalium* in the general population ranges between 1% and 4% [3] but is found more frequently (up to 40%) in risk populations, such as men who have sex with men (MSM), urethritis, and HIV-positive patients, respectively. Eradication of *M. genitalium* is hampered by many asymptomatic cases and problems in treating confirmed infections. Besides the intrinsic resistance to all beta-lactams, the use of doxycycline is of limited clinical efficacy [4]. Thus, current guidelines recommend macrolides (azithromycin) as first choice antibiotics followed by quinolones (moxifloxacin) in cases of therapy failure [5]. Due to the rising occurrence of strains with resistance-associated mutations in 23S rRNA (macrolides) and the *parC* gene (quinolones) worldwide, as well as the lack of effective and approved therapeutic alternatives [6], further epidemiological information about the mechanisms of resistance development and possible correlations between resistance and genotypes of *M. genitalium* is needed.

Whole genome data of a limited number of *M. genitalium* strains showed a high level of recombination in particular regions and low overall nucleotide divergence between genomes [7]. Furthermore, previous studies investigated the usefulness of easy-to-use and comparable approaches to differentiate *M. genitalium* strains directly from PCR-positive clinical samples. These included the analysis of one of the variable regions of the gene coding for the MgpB adhesin (*MG_191*) of the microorganisms and of the variable number of tandem repeats (VNTR) in different regions [8,9]. *MgpB* typing is the most frequently used approach showing a high discrimination power that has led to a relatively great number of more than 100 types characterized to date [10], enabling comparisons of strains occurring in different populations or at different locations. Combination of *mgpB* typing and VNTR in gene *MG_309* was described as useful for the investigation of sexual networks [8,9].

In the present study, we analyzed the *mgpB* types of first and follow-up samples of *M. genitalium*-positive patients in Berlin, Germany, to get an overview of circulating strains in a risk population characterized by MSM and HIV-positives in a metropolitan area. Information about the occurrence of mutations in 23S rRNA and the *parC* gene [11] allows the classification of resistance and types. The results confirmed a great spectrum of *mgpB* types. In addition, *mgpB*/*MG_309* typing is a useful method for comparing first and follow-up samples of patients to distinguish between ongoing colonization with a strain of identical *mgpB*/*MG_309* profile and probable new infection with a different strain. 

## 2. Results

**Figure 1:**
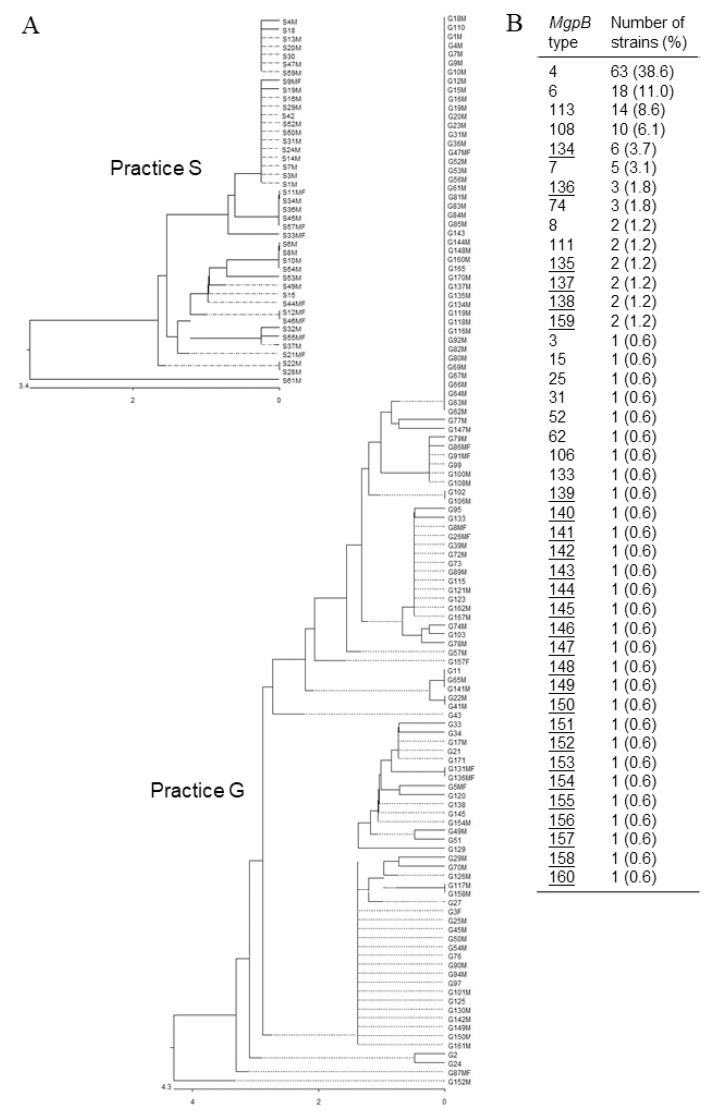
Similarity and distribution of partial *mgpB* sequences of 163 *Mycoplasma genitalium* strains. (**A**) Dendrograms of sequences from samples from practice S and G based on alignment by CLUSTALW (M—strain with macrolide resistance-associated mutation, F—strain with fluoroquinolone resistance-associated mutation). Bar—nucleotide substitution per 100 residues. (**B**) Percentage distribution of the 43 *mgpB* types confirmed in the study (underlined: new types in comparison with types described in Supplementary Table S1).

The *mgpB* type of *M. genitalium* strains was identified in 163 first samples. In Figure 1A, the similarity of sequences of samples obtained from both practices (practice S: n = 43, practice G: n = 120) is summarized in dendrograms. Overall, the occurrence of 43 different *mgpB* types was confirmed, resulting in a discriminatory index of 0.827. Most of the strains (64.4%) belong to four types: 4 (38.6%), 6 (11.0%), 113 (8.6%), and 108 (6.1%), respectively (Figure 1B). Only one strain could be assigned to the predominant number of types (n = 29, 67%). Strains showing mutations associated with macrolide or quinolone resistance were detected in 70% and 30% of types, respectively. However, regarding the four most common *mgpB* types, the rates of resistant strains are different (type 4: 96.8% macrolide and 1.6% quinolone resistance; type 6: 72.2 and 11.1%; type 113: 76.9 and 15.4%; type 108: 90.0 and 30.0%, respectively). In comparison with the 133 *mgpB* types described to date (Supplementary Table S1), 27 new sequences were found. The derived amino acid sequences of the part of the MgpB adhesin (aa 78 to 140) demonstrated 36 differences from the sequence of reference strain G37 (Supplementary Figure S1). For two types (133 and 160), the insertion of two amino acids was confirmed. In comparison to G37, 73% of differences are limited to four amino acids: Ser107 (42 of 43 types detected in the present study), Ser101 (33/43), Asp96 (20/43), and Ala117 (15/43), respectively. Some *mgpB* types showed differences of nucleotide sequences from all other types but are identical with respect to the amino acid sequence (type 3 = types 7, 113, 143; type 4 = 62; type 6 = 153; type 15 = 111, 137, 141).

The *mgpB* types in first and follow-up specimens were compared in 35 patients (Table 1). In seven men, two or more follow-up samples were analyzed. The same *mgpB* type in first and follow-up sample was confirmed in 25 patients (71.4%). The time between samplings varied from 8 (patient no. 34) up to 392 days (patient no. 25). In contrast, specimens from 10 patients (time between samplings: 7 to 324 days) showed different types. Interestingly, among these follow-up samples, further new types were not found. Using VNTR typing of *MG_309* gene as an additional approach for discrimination, the change of *mgpB* type in 8 of 10 cases was combined with different VNTR types of *M. genitalium* in the first and follow-up specimens. Changes of *mgpB* types in two patients (no. 18 and 28) were not associated with alterations of *MG_309* types. Of note, the emergence of mutations associated with quinolone resistance occurred exclusively in follow-up samples that differed in both *mgpB* and VNTR types. Furthermore, a change in macrolide resistance-associated mutations (A to G or A to G/T at position 2071 or 2072 of 23S rRNA) or the occurrence of an SNP of 23S rRNA further suggests an infection of these patients with genetically different strains during the time between samplings. In contrast, mutations of 23S rRNA were detected in follow-up strains of two patients (no. 12 and 30, respectively) without a change in their *mgpB* and VNTR type, supporting the hypothesis that resistance develops during treatment with azithromycin. Among the remaining 33 cases, strains without macrolide resistance-associated mutations in first and follow-up specimens of two patients were demonstrated. In one patient (no. 19), the *mgpB*/*MG_309* profile of the strain in both samples was identical, indicating failure of azithromycin treatment. Typing in a second patient (no. 26) resulted in different *mgpB* and *MG_309* types, making a re-infection with a macrolide-susceptible strain probable. All other patients (89%) carried macrolide-resistant strains in first and follow-up specimens.

## 3. Discussion

In this retrospective study, the *mgpB* types of *M. genitalium* strains from outpatients in Berlin, Germany, were determined. The data give a detailed picture of circulating types in a single location and during a relatively short time period of sixteen months. Furthermore, most of the patients belong to the group of MSM and/or HIV-positives considered as risk populations for infections with this pathogen. In recent studies, the prevalence of *M. genitalium* in both groups ranged between 3% and 17% [3,12,13,14,15]. To our knowledge, this is the first study determining *mgpB* types among patients strongly predominated by MSM. With respect to the 133 types characterized in previous studies (Supplementary Table S1), we confirmed the occurrence of 16 known and 27 new types among 163 patients. Including these new types detected in the present report, a total of 160 *mgpB* types have been described to date, confirming the extent of possible nucleotide changes and insertions in the relatively small part of the *mgpB* gene of around 200 bases. The discriminatory index of the method was calculated to be 0.85 [9], 0.94 [8], and 0.95 [10,16], respectively, and *mgpB* typing was recommended for general studies of *M. genitalium* epidemiology. In previous reports, *mgpB* types were found to be identical in many of the investigated follow-up samples collected over long time periods from the same patient [8,9,16,17,18] and to be stable after a cultural passage of a limited number of isolates [9].

Despite the great diversity of *mgpB* types among the *M. genitalium* strains of the present study, around 50% of all samples belong to two types (four and six, respectively). Both types were first described after investigation of strains in a collection of *M. genitalium*-positive samples from different countries [16]. Remarkably, type four was also found in 32% of samples investigated in a small German study that characterized *M. genitalium* strains between 2015 and 2016 [19]. Belonging of many strains to few *mgpB* types might be caused by relations between patients, like sexual networks, explaining the occurrence of clusters. This suggestion is supported by the relatively low discriminatory index of 0.83 calculated in the present study. It is speculative to decide whether this dominance of particular types is the result of the more probable transmission of frequently occurring strains or of the more virulent properties of some strains. Consistent with the sequence-variable P1 protein of the phylogenetically related species *Mycoplasma pneumoniae* that causes community-acquired infections of the human respiratory tract, the MgpB adhesin of *M. genitalium* can be considered as important for host-pathogen interaction [20]. Great parts of this membrane protein were found as cell surface-exposed, but the near N-terminal region, including the typing sequence, showed no reaction with sera of infected animals, and antibodies to this protein part had no influence on hemadsorption [21]. The occurrence of identical *mgpB* types over long time periods in many follow-up samples of our study and other reports suggests that the host immune system has limited influence on changes in the MgpB typing region during colonization.

Interestingly, the *M. genitalium* strains in samples from the four women included in this report belong to different *mgpB* types (4, 8, 31, and 74, respectively). Because of the low number of women, further studies will have to clarify whether significant differences of *mgpB* types between both gender and between homo- and heterosexual patients in a defined region can be demonstrated.

Here, the typing of *M. genitalium* was performed among samples demonstrating relatively high resistance rates of 79.9% (macrolides) and 13.0% (quinolones), respectively [11]. A clear correlation between distinct *mgpB* types and the occurrence of resistance-associated mutations was not found. Macrolide- and quinolone-resistant strains were confirmed in 30 and 13 of the 43 types determined. Despite the fact that knowledge about the genotype of a clinically relevant strain has had no consequences for therapy up to now, the relations between resistance and typing markers are of epidemiological interest. Regarding the related species *M. pneumoniae*, results of different studies indicated a relationship between the occurrence of macrolide resistance as well as the clinical severity of infections with particular sequence or VNTR type strains [22,23,24]. 

Combination of *mgpB* and *MG_309* typing is suitable to discriminate *M. genitalium* strains colonizing different patients as well as strains in follow-up samples from the same patient [8,9]. In this context, further data about differences of types are important in investigating whether the emergence of resistance-associated mutations is caused by the development of resistance during treatment or by the acquisition of a genetically different strain in the period between samplings [10]. Here, the *mgpB* types of 71% of strains in follow-up samples correspond to the type in the first sample. As expected, differences of type between first and follow-up sample(s) seem to not depend on the time between samplings, as the type remained unchanged in many patients, confirming the long-term stability of this typing marker, as already reported in other studies [8,9,16,17,18]. In contrast, the *mgpB* type of *M. genitalium* in the follow-up specimens from 10 of 35 patients differed in comparison with previous sample(s). This rate is relatively high in comparison with previous investigations, which analyzed mainly strains from heterosexual patients [8,17]. Sexual behaviour (number of contact persons, condom use, participation in sexual networks) can be assumed as important for these differences and should be further investigated in controlled studies. The change of *mgpB* type in the follow-up samples of the present report comes along with differences in the number of VNTR in *MG_309,* supporting the hypothesis of newly acquired infections in these patients with genetically different strains. The occurrence of mutations associated with quinolone resistance in the follow-up samples of three patients was linked with a change of *mgpB* type and a different number of repeats in *MG_309*. Importantly, quinolones were not prescribed between sampling. Conversely, the development of macrolide resistance in the follow-up specimens of two patients was combined with an identical *mgpB*/*MG_309* profile, supporting the emergence of resistance during therapy with azithromycin [10,11]. It should be noted that the colonization of a patient with strains showing different *mgpB* types cannot be excluded. In the present study, two locations (urethra and rectum) of four patients were sampled simultaneously. In one case, the *mgpB* types differed (data not shown), which complicates the evaluation of the results of typing and may explain differences between samples to some extent.

In conclusion, the results of the present study confirm a great diversity of *mgpB* types among MSM in Berlin, Germany. A clear correlation of the type with macrolide and quinolone resistance-associated mutations was not found. However, *mgpB* typing is useful for monitoring the circulation of strains among different populations, as well as in combination with VNTR characterization in *MG_309*, to compare first and follow-up samples from patients. Thus, typing of *M. genitalium* strains is a valuable and reliable tool for studies to further understand the epidemiology, treatment failures, and development of antimicrobial resistance of an emerging sexually transmitted pathogen.

## 4. Material and Methods

Primarily, to test the prevalence of macrolide and quinolone resistance [11], samples investigated in this study were collected in Berlin, Germany, between September 2017 and December 2018 in two practices (designated as S and G) specialized in the treatment of STIs. The included patients are predominately MSM with a relatively high portion of HIV-positives (Table 2). The retrospective study was approved by the Institutional Review Board of the TU Dresden (no.: EK 473122017).

As described recently [11], the DNA of the samples was extracted by the EZ1 DNA tissue kit and EZ1 Advanced XL (Qiagen) automated extraction system and tested for *M. genitalium* by real-time PCR using a commercial assay (Anyplex^TM^ STI-5 Detection Assay; Seegene). DNA of positive samples was stored at −20 °C until the determination of genotypes. Overall, 163 *M. genitalium*-positive first specimens from 163 different patients were included in the present study. In addition, 45 follow-up samples of 35 different patients were investigated. All samples of the study were pretested regarding the occurrence of resistance-associated mutations in the 23S rRNA (macrolides) and *parC* gene (quinolones) of *M. genitalium* [11].

*MgpB* types were determined after amplification of the typing region (nt 180–460) of *MG_191* gene by a nested PCR approach (35 cycles each; Eppendorf personal cycler) and sequencing as described [19]. PCR products were purified using the MSB Spin PCRapace kit (Stratec). Nucleotide and derived amino acid sequences were compared with the corresponding part of the reference genome of type strain G37 (GenBank accession no. NC_000908.2) and with the 133 *mgpB* types published to date (Supplementary Table S1). The discriminatory index of *mgpB* typing was calculated as reported in [25]. Phylogenetic tree prediction was performed by using CLUSTALW (DNASTAR lasergene).

To further discriminate first and follow-up samples, the variable number of tandem repeats in the *MG_309* gene of *M. genitalium* was identified as reported in [19].

The sequences of 27 new *mgpB* types described in this study were deposited in GenBank (accession numbers: MN387712-MN387714, MN387716-MN387739).

## Figures and Tables

**Table 1:** Comparison of *mgpB* and *MG_309* genotypes of *M. genitalium* in first and follow-up samples from the same patient (grey highlighted: difference between *mgpB* and/or *MG_309* types).

Patient no.	Samples	Time Between Samples (d)	*MgpB* type^1^	*MG_309* type^2^	Profile (*mgpB*+*MG_309*)	Comments
1	S7/S17	29	4	9/9	4-9	
2	S10/S27	41	134	10/10	134-10	
3	S11/S23	30	108	n.d.^3^	-	
4	S12/S25	27	12	n.d.	-	
5	S36/S48	23	108	10/10	108-10	
6	S45/S56	35	108	10/10	108-10	
7	G1/G14	27	4	12/12	4-12	
8	G4/G32	48	4	10/10	4-10	
9	G5/G28	43	145	11/11	145-11	
10	G8/G46	81	113	9/9	113-9	
11	G9/G60	85	4	12/12	4-12	
12	G11/G35	27	7	11/11	7-11	development of MRAM^4^
13	G12/G42	42	4	8/8	4-8	
14	G17/G40/G68	30/46	133/133/137	10/10/11	133-10/137-11	
15	G18/G44	36	4	10/10	4-10	
16	G20/G30	12	4	10/10	4-10	
17	G22/G48/G71	49/56	138	12/12/12	138-12	
18	G25/G55	53	6/136	11/11	6-11/136-11	difference in MRAM, emergence of QRAM^5^
19	G43/G58	50	136	13/13	136-13	
20	G56/G93	166	4/136	9/13	4-9/136-13	difference in MRAM, emergence of QRAM
21	G70/G164	324	151/7	11/10	151-11/7-10	difference in MRAM
22	G80/G111/G122	154/24	4/134/134	9/10/10	4-9/134-10	SNP A2200G of 23S rRNA between G80 and G111
23	G81/G98	97	4	11/11	4-11	
24	G86/G107	96	152/4	11/10	152-11/4-10	difference in MRAM
25	G91/G114/G128/	91/64/	108	10/10/10/	108-10	
	G168/G204	87/150		10/10		
26	G99/G104	14	108/113	9/11	108-9/113-11	
27	G101/G105	7	6/108	11/10	6-11/108-10	
28	G106/G113	36	137/4	10/10	137-10/4-10	
29	G121/G124	13	113	14/14	113-14	
30	G115/G127	35	113	11/11	113-11	development of MRAM
31	G130/G132/G156	11/63	6/6/7	11/11/9	6-11/7-9	difference in MRAM, emergence of QRAM
32	G131/G140	21	159	9/9	159-9	
33	G147/G166/G174	39/59	4	9/9/9	4-9	
34	G161/G163	8	6	11/11	6-11	
35	G154/G169/	48/91/	2	9/9/9/9	2-9	
	G186/G202	50				

1—Description according to supplementary Table S1; 2—number of tandem repeats in MG_309; 3—not determined (sequencing results not evaluable); 4—MRAM: macrolide resistance-associated mutation.; 5—QRAM: quinolone resistance-associated mutation.

**Table 2:** Characteristics of *M. genitalium*-positive patients (n = 163) and specimens included in the study.

Variable	
Mean age (years; range)	36.1 (20–61)
Males (%)	97.5
MSM (%)	92.6
HIV-positive (%)	46.6
First specimens	
Rectal swabs (%)	57.7
First-void urine (%)	38.6
Vaginal swabs (%)	2.4
Urethral swab (%)	0.6
Pharyngeal swab (%)	0.6
Follow-up specimens (n = 45)	
Number of patients (% of all patients)	35 (21.5)
Rectal swabs (% of follow-up specimens)	30 (66.7)
First-void urine (%)	14 (31.1)
Urethral swab (%)	1 (2.2)

## Supplementary Materials

The following are available online at https://www.mdpi.com/2076-0817/9/1/12/s1, Table S1: Designation of types of M. genitalium strains based on mgpB typing. Sequences described in supplemental material of the publications (1) or deposited in GenBank (2–11), as well as the types found in the present study, were aligned with MegAlign (DNASTAR lasergene), Figure S1: Alignment of partial MgpB amino acid sequences of type strain G37 and of the 43 mgpB types detected in the present study. Positions according to the sequence of G37. For designation of types see supplementary Table S1.
