# Supplementary material for: MgpB Types among Mycoplasma genitalium Strains from Men Who Have Sex with Men in Berlin, Germany, 2016–2018
Source: Pathogens. 2019 Dec 20;9(1):12. doi: 10.3390/pathogens9010012 (PMC7168617; doi:10.3390/pathogens9010012)

Supplemental material

Table S1. Designation of types of *M. genitalium* strains based on *mgpB* typing. Sequences described in supplemental material of the publications (1) or deposited in GenBank (2-11) as well the types found in the present study were aligned with MegAlign (DNASTAR lasergene).

**Proposed type description**	**Reference**
1 - 56	(1)
57 - 60	(2)
61 - 74	(3)
75 - 78	(4)
79 - 86	(5)
87 - 89	(6)
90 - 99	(7)
100	(8)
101 - 105	(9)
106 - 111	(10)
112 - 133	(11)
134 - 160	present study

References

1. Hjorth, S.V.; Björnelius, E.; Lidbrink, P.; Falk, L.; Dohn, B.; Berthelsen, L.; Ma, L.; Martin, D.H.; Jensen, J.S. Sequence-based typing of *Mycoplasma genitalium* reveals sexual transmission. *J. Clin. Microbiol.* **2006**, *44*, 2078-2083.
2. Ma, L.; Taylor, S.; Jensen, J.S.; Myers, L.; Lillis, R.; Martin, D.H. Short tandem

repeat sequences in the *Mycoplasma genitalium* genome and their use in a multilocus genotyping system. *BMC Microbiol.* **2008**, *8*, 130.

3. Musatovova, O; Baseman, J.B. Analysis identifying common and distinct sequences among Texas clinical strains of *Mycoplasma genitalium*. *J. Clin. Microbiol.* **2009**, *47*, 1469-1475.
4. Iverson-Cabral, S.L.; Astete, S.G.; Cohen, C.R.; Rocha, E.P.; Totten, P.A. Intrastrain heterogeneity of the mgpB gene in *Mycoplasma genitalium* is extensive in vitro and in vivo and suggests that variation is generated via recombination with repetitive chromosomal sequences. *Infect. Immun.* **2006**, *74*, 3715-3726.
5. Cazanave, C.; Charron, A.; Renaudin, H.; Bébéar, C. Method comparison for molecular typing of French and Tunisian *Mycoplasma genitalium*-positive specimens. *J. Med. Microbiol.* **2012**, *61*, 500-506.
6. Chrisment, D.; Charron, A.; Cazanave, C.; Pereyre, S.; Bébéar, C. Detection of macrolide resistance in *Mycoplasma genitalium* in France. *J Antimicrob Chemother*. **2012**, *67*, 2598-2601.
7. Pond, M.J.; Nori, A.V.; Witney, A.A.; Lopeman, R.C.; Butcher, P.D.; Sadiq, S.T. High prevalence of antibiotic-resistant *Mycoplasma genitalium* in nongonococcal urethritis: the need for routine testing and the inadequacy of current treatment options. *Clin. Infect. Dis.* **2014**, *58*, 631-637.
8. Mondeja, B.A.; Jensen, J.S.; Rodríguez, I.; Morier, L.F.; Kourí, V.; Rodríguez, N.M.; Fernández, C. Isolation of *Mycoplasma genitalium* from patients with urogenital infections: first report from the Latin-American region. *New Microbes New Infect.* **2013**, *1*, 22-26.

9. Kikuchi, M.; Ito, S.; Yasuda, M.; Tsuchiya, T.; Hatazaki, K.; Takanashi, M.; Ezaki, T.; Deguchi, T. Remarkable increase in fluoroquinolone-resistant *Mycoplasma genitalium* in Japan. *J. Antimicrob. Chemother.* **2014**, *69*, 2376-2382.
10. Dumke, R.; Thürmer, A.; Jacobs, E. Emergence of *Mycoplasma genitalium* strains showing mutations associated with macrolide and fluoroquinolone resistance in the region Dresden, Germany. *Diagn. Microbiol. Infect. Dis.* **2016**, *86*, 221-223.
11. Piñeiro, L.; Idigoras, P.; Cilla, G. Molecular typing of *Mycoplasma genitalium*-positive specimens discriminates between persistent and recurrent infections in cases of treatment failure and supports contact tracing. *Microorganisms*. **2019**, *7*, 609. doi: 10.3390/microorganisms7120609.

Figure S1 Alignment of partial MgpB amino acid sequences of type strain G37 and of the 43 *mgpB* types detected in the present study. Positions according to sequence of G37. For designation of types see supplementary table 1.

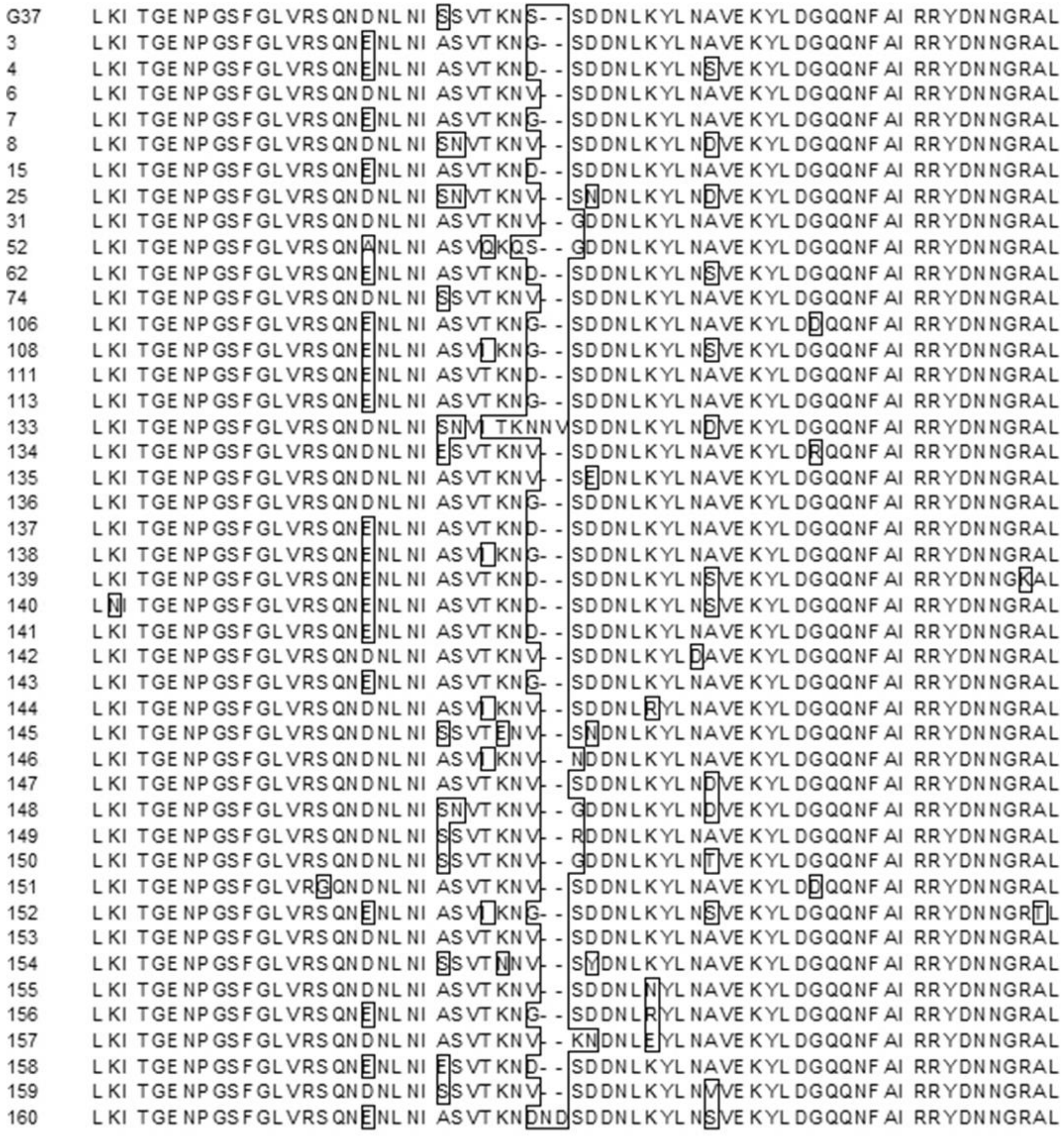

Supplement: Supplementary file 1 [file pathogens-09-00012-s001.pdf]
